# 2943. Hazardous Alcohol Use and Direct-acting Antiviral Therapy Initiation Among People Who Inject Drugs with Current Hepatitis C Infection

**DOI:** 10.1093/ofid/ofad500.182

**Published:** 2023-11-27

**Authors:** Hamidreza Karimi-Sari, Oluwaseun Falade-Nwulia, Katie Zook, Kathleen R Page, Gregory Lucas

**Affiliations:** Division of Infectious Diseases, Johns Hopkins University School of Medicine, Baltimore, Maryland, USA, Baltimore, MD; Johns Hopkins University, Baltimore, MD; Johns Hopkins University, Baltimore, MD; Johns Hopkins School of Medicine, Baltimore, MD; Division of Infectious Diseases, Johns Hopkins University School of Medicine, Baltimore, Maryland

## Abstract

**Background:**

People who inject drugs (PWID) have a high prevalence of hepatitis C virus (HCV) infection. However, HCV treatment rates among PWID remain low. Previous data suggest that hazardous alcohol use is negatively associated with direct-acting antiviral (DAA) therapy initiation in the general population. We aimed to evaluate the effect of hazardous alcohol use on DAA therapy initiation among PWID.

**Methods:**

PWID were recruited through street outreach in Baltimore, Maryland 2018–2019. Participants completed a study survey and HCV testing (antibody reflex to RNA) at study visits. DAA initiation (self-report) was assessed at study follow-up in 6-month increments. Hazardous alcohol use was defined as Alcohol Use Disorders Identification Test-C (AUDIT-C) ≥4 for men or ≥3 for women. Frequency of injection drug use was categorized as ≤3 vs. >3 times/day. Longitudinal data were analyzed using descriptive statistics and multivariable logistic regression analyses with generalized estimating equations (GEE).

**Results:**

Among 720 PWID, 291 (40.4%) had current HCV infection evidenced by detectable HCV-RNA. Among 134 people with HCV viremia and awareness of their HCV infection, the mean age (± standard deviation) was 48.7 ± 10.3 years, 53% were males, 64.9% were African-American, 59.7% had hazardous alcohol use and 60.4% reported >3 injections per day. DAA therapy was initiated in 16 (11.9%) PWID within 6 months (Table 1). In univariate analysis, DAA treatment was not associated with hazardous alcohol use (OR=1.84, 95%CI=.67–5.06) or injection frequency (OR=.54, 95%CI=.21–1.40) but was associated with Black race (OR=4.48, 95%CI=1.27–15.73) and age ≥50 years old (OR=7.17, 95%CI=2.04–25.26). In multivariate analysis, hazardous alcohol use (aOR=1.20, 95%CI=0.41–3.48), injection frequency (aOR=.84, 95%CI=.32–2.24), and Black race (aOR=1.88, 95%CI=.64–5.55) were not associated with DAA treatment initiation. Older age ≥50 vs. < 50 years was positively associated with HCV treatment initiation (aOR=4.79, 95%CI=1.61–14.22).

**Figure:**
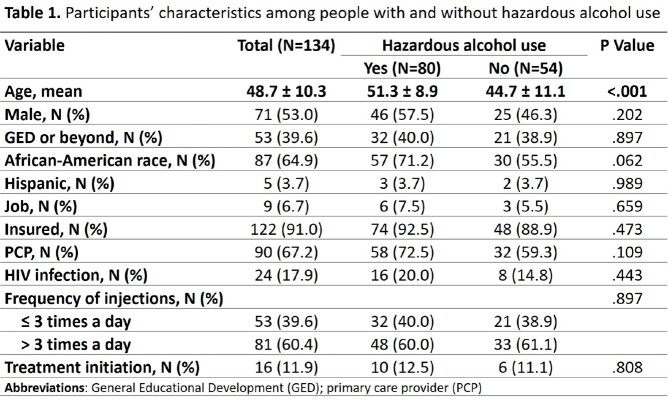

**Conclusion:**

Among our sample of PWID with HCV infection, oral DAA treatment was not associated with self-reported hazardous alcohol use. The overall very low rates of HCV treatment are concerning and need to be addressed by effective interventions, especially for younger PWID.

**Disclosures:**

**Oluwaseun Falade-Nwulia, MBBS ,MPH**, Abbvie Inc: Grant/Research Support|Gilead Sciences: Advisor/Consultant

